# *miR-34a* Regulates Lipid Droplet Deposition in 3T3-L1 and C2C12 Cells by Targeting LEF1

**DOI:** 10.3390/cells12010167

**Published:** 2022-12-30

**Authors:** Lixue Wang, Yuhuai Xie, Wei Chen, Yu Zhang, Yongqing Zeng

**Affiliations:** 1 Shandong Provincial Key Laboratory of Animal Biotechnology and Disease Control and Prevention, College of Animal Science and Technology, Shandong Agricultural University, Tai’an 271018, China; 2 School of Medicine, Huanghe Science and Technology College, Zhengzhou 450063, China; 3 Department of Immunology, School of Basic Medical Sciences, Fudan University, Shanghai 200032, China

**Keywords:** *miR-34a*, *Lef1*, intramuscular fat deposition, adipocytes, muscle cells, adipogenesis

## Abstract

Intramuscular fat (IMF) content plays a key role in improving the flavor and palatability of pork. The IMF content varies between species, breeds, and individuals of the same breed. Hence, it is necessary to elucidate the mechanisms of IMF deposition to improve pork quality. Herein, the IMF content in the longissimus dorsi muscles of 29 Laiwu pigs was detected and divided into two groups, the H group (IMF > 12%) and the L group (IMF < 5%). RNA sequencing analysis showed 24 differentially expressed (DE) miRNA, and GO and KEGG analysis demonstrated that the DE miRNAs were significantly enriched in lipid metabolic process, lipid storage, Wnt, mTOR, and PPAR signaling pathways. *miR-34a* was found to be increased in the H group and 3T3-L1-derived adipocytes, while *Lef1* was decreased. Luciferase reporter assays demonstrated that *Lef1* was a potential target of *miR-34a*. Mechanism analysis revealed that *miR-34a* could increase lipid droplet deposition in 3T3-L1 and C2C12 cells by dampening the suppressive function of Lef1 on the transcription of adipogenic markers (i.e., *Pparg*, *Cebpa*, *Fabp4,* and *Plin1*). Moreover, overexpression of *miR-34a* could enhance the lipid deposition in the co-culture system of 3T3-L1 and C2C12 cells as well as in C2C12 cells cultured with conditioned medium from the progress of adipocyte differentiation. Taken together, our study indicated that *miR-34a* was an important positive modulator in the regulation of fatty metabolism and fat deposition by inhibiting the suppressive function of *Lef1*. These results might provide insight for the exploration of potential strategies to promote intramuscular fat deposition in livestock.

## 1. Introduction

Intramuscular fat (IMF) is an essential index of pork quality evaluation, such as tenderness, juiciness, and flavor level [1]. Many factors affect the IMF content of pork, including breed, nutrition, and environment, which ultimately regulate fat deposition by increasing adipocyte proliferation and hypertrophy [2,3]. IMF deposition occurs both inside (intramyocellular) and outside (extramyocellular) the muscle fibers, including the lipid droplets in myoblasts and adipocytes. This biological process is complex and particular. Therefore, the mechanism of IMF accumulation remains a mystery. Evidence has shown that myoblasts can be induced to adipocyte differentiation by means of transcriptional and nutritional factors [4]. Daidzein promotes FAs-induced fat deposition through G-protein-coupled receptor 30 (GPR30) signaling in C2C12 myoblast cells [5]. miR-324-5p inhibits C2C12 myoblast differentiation and promotes lipid deposition in myotubes by targeting long non-coding Dum (lncDum) and peptidase M20 domain containing 1 ang(Pm20d1), respectively [6]. Therefore, the interaction between myoblasts and adipocytes should not be ignored in the process of exploring the mechanisms of IMF deposition. The more interesting finding is that lipid accumulation is heterogeneous within the same cell population [7]. A hypothesis could be established that some specific genes may cause differentiation in lipid droplet deposition in myoblasts or adipocytes.

microRNAs (miRNAs) are a class of endogenous single-stranded highly conserved noncoding RNAs (ncRNAs) with a length of 18–22 nt. The seed sequence of miRNAs is the second to eighth nucleotide starting from the 5′ end, which is an important structure for miRNAs to target mRNAs [8]. In addition, similarity in seed sequence is the basis for miRNA family differentiation [9]. The specific sequence of the 3′-untranslated region (3′UTR) of the target gene is complementary to the miRNA seed sequence and binds to the Argonaute (AGO) protein-silencing complex through guidance to inhibit the translation process of the target gene [10]. Therefore, these previous studies demonstrated that most miRNAs interact with mRNAs in a negative correlation at the post-transcriptional level. Further research on miRNA found that the interaction between miRNAs and mRNAs is not static and changeless but rather involves dynamic and ordered biological processes [11]. The evidence shows that miRNAs are also regulated by a changed cellular environment, and this process also depends on mRNA translation [11,12]. In 2007, it was first reported that miR-369 regulated target mRNAs in two ways, depending on whether the cell medium contained serum [13].

Studies have shown that miRNAs are involved in regulating various biological processes associated with IMF deposition, including adipocyte proliferation and differentiation and lipid metabolism [14,15,16,17]. For instance, miR-375 overexpression can promote 3T3-L1 cell differentiation by reducing the ERK1/2 level and increasing the expression of *Ppar*, *Cebpa*, *Fabp4,* and triglycerides [14]. This miRNA also can promote *Pparg* and *Cebpa* levels by targeting bone morphogenetic protein receptor type 2 (BMPR2) expression to regulate the differentiation process of pork-derived adipocytes [15]. miR-16-5p has also been shown to promote 3T3-L1 cell differentiation through regulating ethanolamine phosphotransferase 1 (EPT1) [16]. In addition, evidence has shown that miRNAs can regulate lipid droplet accumulation in myoblasts. Myostatin can inhibit the expression of glucocorticoid receptor (GR) by activating miR-124-3p and thus regulates IMF deposition [17]. Overexpression of miR-324-5p significantly promoted oleate-induced lipid accumulation and β-oxidation in C2C12 myoblasts by targeting Pm20d1 [6]. It can be inferred from this evidence that miRNAs play an essential role in regulating fat deposition, especially IMF accumulation.

Lymphocyte enhancer factor-1 (Lef1) is a member of the Lef1/T-cell factor (TCF) family and has no transcriptional activation potential by itself, but it can act as an architectural protein in the assembly of multiprotein enhancer complexes [18]. Lef1 could mediate a nuclear response to the canonical Wnt signaling pathway by interacting with β-catenin to regulate adipogenesis [19]. Among the numerous target genes of *miR-34a*, Sirtuin 1 (SIRT1) is the most studied to explore the function of *miR-34a* [20]. Downregulation of *miR-34a* potentially contributes to altered lipid metabolism in nonalcoholic fatty liver disease (NAFLD) by regulating its targets, PPARα and SIRT1 [21]. However, the relationship between *miR-34a* and *Lef1* and the function of these two genes in IMF deposition remain unclear.

The Laiwu pig is an excellent North Chinese breed of pig with a high IMF content. However, it was discovered in pork production that the IMF content varies widely between individual Laiwu pigs [22,23], which has so far been ignored in exploring the mechanisms of IMF deposition. In this study, the results of whole transcriptome sequencing showed that *miR-34a* and *Lef1* are associated with IMF deposition. Additionally, *miR-34a* promoted lipid droplet accumulation in 3T3-L1 and C2C12 cells by targeting *Lef1* to regulate IMF deposition, which might result in the difference in IMF content among individual Laiwu pigs.

## 2. Materials and Methods

### 2.1. Animal Experiments

Twenty-nine 300-day-old male (approximately 96 ± 4 kg) Laiwu pigs, reared at the Laiwu pig breeding farm in Jinan, Shandong Province, China, were fed a commercial pig diet and water ad libitum. Longissimus dorsi (LD) muscle samples from the last rib were obtained immediately after exsanguination and then stored in liquid nitrogen. The samples were stored at −80 °C for RNA and protein analysis. The IMF contents, grouping, and sample selection criteria for the sample groups for whole transcriptome sequencing have been reported in our previous study [23]. In brief, the IMF contents of LD muscle samples from the 29 Laiwu pigs were determined. Then, eight half-sibs of Laiwu pigs were selected and divided into two groups: the high IMF content group (IMF > 12%, in terms of H) and the low IMF content group (IMF < 5%, in terms of L), respectively, according to the IMF content and genetic relationship. The IMF contents in group H are 12.45%, 13.93%, 13.27%, and 13.38%, while the IMF contents in L group are 4.23%, 4.29%, 4.31% and 3.23%. The H group had significantly higher IMF content than the L group (*p* < 0.05). Oil Red O staining was performed and demonstrated the high content of IMF in the H group compared with that in the L group in the previous study, which was reported in our previous study [23]. In addition, three samples (1 × 1 × 1 cm) form heart, liver, spleen, lung, kidney, and subcutaneous fat were collected and immediately snap-frozen in liquid nitrogen. All samples were stored at −80 °C before RNA analysis.

### 2.2. RNA Isolation, Library Preparation, and Sequencing Analysis

Total RNA was isolated from eight LD muscle samples using TRIzol Reagent (Invitrogen, Carlsbad, CA, USA) according to the manufacturer’s recommendations. The RNA purity, concentration, and integrity were measured using the NanoPhotometer^®^ spectrophotometer (IMPLEN, Carlsbad, CA, USA), Qubit^®^ RNA Assay Kit in Qubit^®^ 2.0 Flurometer (Life Technologies, Carlsbad, CA, USA), and the RNA Nano 6000 Assay Kit of the Agilent Bioanalyzer 2100 system (Agilent Technologies, Carlsbad, CA, USA). A total of 3 μg of total RNA per sample was used as the input material for the small RNA library. Sequencing libraries were generated using NEBNext^®^ Multiplex Small RNA Library Prep Set for Illumina^®^ (NEB, Beverley, MA, USA) following the manufacturer’s recommendations and index codes were added to attribute sequences to each sample. Raw sequence data were submitted to the NCBI Sequence Read Archive under accession number PRJNA769962.

The library preparations were sequenced on an Illumina Hiseq 2500/2000 platform and 50 bp Data analysis (Novogene Gene Regulation Department). Raw data (raw reads) in fastq format were first processed through custom Perl and Python scripts. At the same time, Q20, Q30, and GC content of the raw data were calculated. The small RNA tags were mapped to the reference sequence by Bowtie [24] without mismatch to analyze their expression and distribution on the reference. Mapped small RNA tags were used to look for known miRNA. miRNA expression levels were estimated in transcripts per million (TPM), and differentially expressed miRNAs (DE miRNAs) was determined with Padj ≤ 0.05, and |Log_2_ FC| ≥ 0.

### 2.3. Gene Ontology (GO) and Kyoto Encyclopedia of Genes and Genomes (KEGG) Enrichment Analyses

GO enrichment analysis was used on the target gene candidates of DEMs, “target gene candidates”, in the following. GOseq-based Wallenius non-central hypergeometric distribution [25], which could adjust for gene length bias, was implemented for GO enrichment analysis. KEGG [26] is a database resource for understanding high-level functions and utilities of biological systems, such as the cell, the organism, and the ecosystem, from molecular-level information, especially large-scale molecular datasets generated by genome sequencing and other high-throughput experimental technologies (http://www.genome.jp/kegg/, accessed on 1 October 2020). We used KOBAS [27] software to test the statistical enrichment of the target gene candidates in KEGG pathways.

### 2.4. Gene and Protein Expression Analyses

microRNAs were isolated using the miRcute miRNA isolation kit (DP501, Tiangen Biotech, Beijing, China). Levels of *miR-34a* were determined by qPCR (FP411, Tiangen Biotech, Beijing, China). Total RNA was isolated with TRIzol and determined by qPCR (AG11701, Accurate Biotechnology, Hunan, China). Primer sequences are available in Table 1. All experiments were conducted at least three times. Relative gene expression was calculated by the 2^−ΔΔ Cq^ method using β-actin and U6 as the housekeeping genes. For Western blot, total protein was extracted using radioimmunoprecipitation assay (RIPA) buffer (Beyotime, Shanghai, China). A total of 20 μg of protein was separated by SDS-PAGE and transferred to polyvinylidene fluoride (PVDF) membranes (Solarbio, Beijing, China). Membranes were then incubated overnight with primary antibodies as follows: anti-LEF1 (1:1000, ab137872, Abcam, Cambridge, MA, USA), anti-C/EBPα (1:1000, ab40764, Abcam, Cambridge, MA, USA), anti-PPARγ (1:1000, ab272718, Abcam, Cambridge, MA, USA), anti-β-catenin (1:1000, ab32572, Abcam, USA), and GAPDH (1:1000, AF0006, Beyotime, Shanghai, China). All antibodies were diluted using Western dilution buffer (P0023, AF0006, Beyotime, Shanghai, China). The next day, the membranes were washed three times with Tris-buffered saline containing Tween 20 (TBST). Then, the membranes were incubated with a goat anti-rabbit IgG (1:1000, A0208, Beyotime, Shanghai, China), or goat anti-mouse IgG (1:1000, A0216, Beyotime, Shanghai, China) for 1 h at 4 °C with shaking. Finally, the protein bands were visualized through an enhanced chemiluminescence (ECL) reagents (Beyotime, China), and the band intensities were quantified using a gel imager system (Alpha Innotech, San Leandro, CA, USA). The intensities of all the immunoblot bands were normalized to those of the corresponding internal standard bands (GAPDH).

### 2.5. Cell Culture and Treatments

The 3T3-L1, C2C12, and HEK293 cell lines (Procell, Wuhan, China) were cultured in a humidified CO2 chamber at 37 °C using DMEM (Gibco, GrandIsland, NY, USA) containing penicillin, streptomycin (Invitrogen, Carlsbad, CA, USA), and 10% fetal bovine serum (FBS) (Gibco, GrandIsland, NY, USA) (this complete medium was considered as M1). Cells were regularly subcultured before reaching 70% confluence, and the passage number was less than eight.

3T3-L1 cells were plated on a 100 mm culture dish at a very high density (3–4 million in 20 mL medium) and incubated overnight. On the second day (d 0), the medium was gently replaced with stage I differentiation medium of M1 medium supplemented with 10 mg/L insulin (Fosun Pharma, Shanghai, China), 1 μM dexamethasone (DEX, Solarbio, Beijing, China), and 0.5 mM 3-isobutyl-1-methylxanthine (IBMX, Solarbio, Beijing, China). The medium was switched to DMEM with 10% FBS and insulin only on d 2. Insulin was removed from the medium, and cells were maintained in M1 medium from d 4, changing the medium every 2 days.

For the myogenic differentiation, C2C12 cells were induced with DMEM supplemented with 2% horse serum after reaching the contact inhibition stage. The culture medium was changed every 2 days. After 6 days of C2C12 differentiation, when the myotubes were visible, the cells were incubated with DMEM containing 0.5% FBS and 500 μM oleate for 24 h to achieve the induction of lipid deposition in myotubes [28].

**Figure 1:**
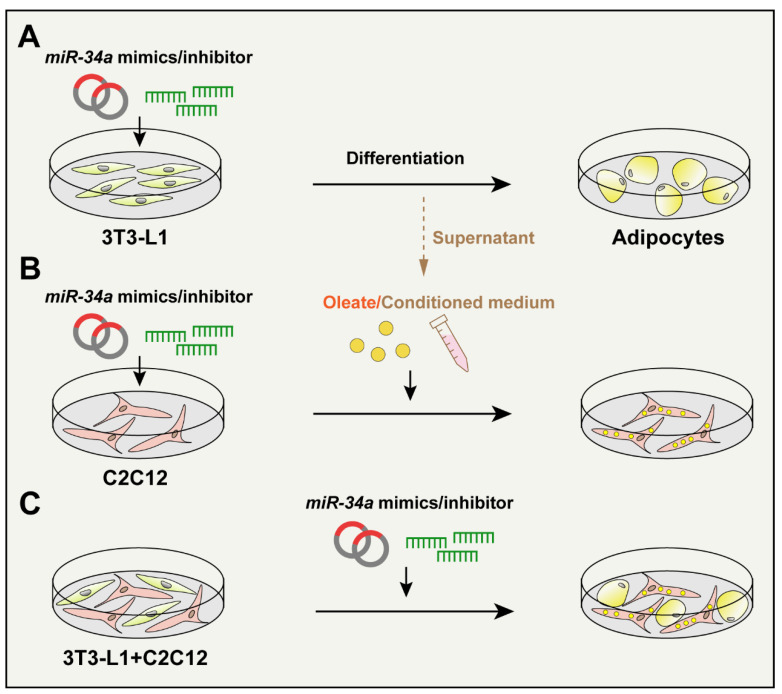
Cell culture and treatments. (**A**) 3T3-L1 cells were treated with *miR-34a* mimics or inhibitor (siRNA) and then were induced by adipocyte differentiation medium. (**B**) C2C12 cells were pre-treated by *miR-34a* mimics or inhibitor (siRNA) and treated with oleate or conditioned medium from the differentiation progress of 3T3-L1-derived adipocyte medium. The expression of adipogenic markers (i.e., *Pparg*, *Cebpa*, *Fabp4,* and *Plin1*) and the lipid droplet deposition in C2C12 cells were detected. (**C**) 3T3-L1 and C2C12 cells were co-cultured and treated with *miR-34a* mimics or inhibitor (siRNA) to detect the expression of adipogenic markers and lipid droplet deposition.

In order to determine the mechanisms of *miR-34a*/*Lef1* function in lipid droplet accumulation in adipocytes and myoblasts, the particularity and complexity of the IMF internal microenvironment were fully considered. Cell treatments were as follows (Figure 1): (1) exploring the function of *miR-34a*/*Lef1* in the differentiation process of preadipocytes (3T3-L1 cells); (2) determining the regulation of *miR-34a*/*Lef1* in the lipid droplet deposition in myoblasts (C2C12 cells); (3) detecting the effects of *miR-34a*/*Lef1* on fat accumulation in co-cultured 3T3-L1 and C2C12 cells; and (4) after *miR-34a* was overexpressed, the culture medium of preadipocytes during differentiation was collected to prepare the conditioned medium. C2C12 cells were cultured in the conditioned medium for 48 h after inducing C2C12 cells for myoblast differentiation to explore the effect of the conditioned medium of adipocytes on lipid droplet deposition in C2C12 cells, which was based on a similar study performed on macrophages [29].

### 2.6. Lef1 and miR-34a Mimics and Silencing

*miR-34a* mimics, a *miR-34a* inhibitor, and their respective negative controls (NC) were synthesized by GenePharma (Shanghai, China). Sequences are listed in Table 1. A *Lef1* sequence was synthesized and subcloned into a transfection plasmid to generate recombinant vector pcDNA3.1-*Lef1* (described as *Lef1* in Figure 1). The control group was transfected with an empty pcDNA3.1 plasmid (described as pcDNA3.1 in Figure 1). We purchased specific siRNAs for mouse *Lef1* from GenePharma. Plasmid and siRNA were transfected into cells using Lipofectamine™ 2000 (Invitrogen, Carlsbad, CA, USA) according to the manufacturer’s instructions.

### 2.7. Oil Red O Staining

Cultured cells were tested for lipid droplet formation using Oil Red O staining. Cells were washed in PBS and then fixed in 4% paraformaldehyde (Solarbio, Beijing, China) for 20 min. Then, the cells were washed briefly in 60% isopropanol (Hushi, Shanghai, China) and stained with 0.5 g Oil Red O (Solarbio, Beijing, China) stain for 30 min. Stained cells were washed in distilled water. Samples were then counterstained with hematoxylin (Solarbio, Beijing, China) for 1 min and then observed under a bright microscope (Nikon Eclipse Ni-U; Nikon, Tokyo, Japan), and images were taken with Nikon Digit sight DS-Fi2 digital camera (Nikon, Tokyo, Japan) and analyzed using image-analyzing program NIS Elements BR (Version 4.20). Then, the cells were treated with 1 mL isopropanol to extract the Oil Red O, followed by OD value detection at 510 nm [28].

### 2.8. RNA Fluorescence In Situ Hybridization (RNA FISH)

SA-Cy3-labeled *miR-34a* probes were obtained from GenePharma (Shanghai, China). RNA FISH was performed using a fluorescent in situ hybridization kit (GenePharma) following the manufacturer’s instructions.

### 2.9. BODIPY Staining

Cells were washed three times with PBS and fixed in 4% paraformaldehyde for 30 min. After washing three times with PBS, BODIPY (MK biotechnology, Shanghai, China) staining was performed for 30 min. Cells were then washed three times (5 min each wash) with PBS, with shaking. Finally, cells were stained with DAPI for 10 min, washed three times, and then imaged under a fluorescence microscope.

### 2.10. Immunofluorescence Staining

Cells were fixed in 4% paraformaldehyde for 30 min at room temperature, washed three times with PBS, and permeabilized with 0.1% Trition X-100 (Solarbio, Beijing, China) for 10 min. After blocking with 2% BSA (Gibco, GrandIsland, NY, USA) in PBS for 2 h at room temperature, the cells were washed three times with PBS and then incubated overnight at 4 °C with LEF1 antibody (1:100 dilution, ab137872, Abcam, Cambridge, MA, USA) or MyHC antibody (1:100 dilution, ABclonal, Wuhan, China). The next day, the cells were washed three times with PBS, 10 min each wash, and subsequently incubated with secondary antibodies (FITC-labeled Goat Anti-Rabbit IgG (H + L), Beyotime, Shanghai, China and Alexa Fluor 555-conjugated Goat Anti-Rabbit IgG (H + L), ABclonal, Wuhan, China) for 2 h at room temperature, and then washed with PBS. Finally, the cells were counterstained with DAPI (Beyotime, Shanghai, China) and viewed on a Nikon.

### 2.11. Dual Luciferase Reporter Assay

A dual-luciferase reporter assay was employed to examine the binding activity between *miR-34a* and *Lef1* following established protocols in HEK293T cells [30]. First, we constructed dual-luciferase reporter gene plasmids ligated to both wild-type and mutant forms of *Lef1* gene coding sequences. In brief, HEK293T cells from all groups were transfected with a pGLO-Luc plasmid containing candidate binding elements, including *Lef1* gene fragments linked to a firefly luciferase neo expression vector, using the approaches mentioned above. After transfection, HEK293T cells were measured for Renilla luciferase activity using the Dual-Luciferase Assay Kit (Promega, Madison, WI, USA) according to the manufacturer’s instructions. Firefly and Renilla luciferase activities were measured by a Full Function Detector (PerkinElmer/envision) and normalized to Renilla luciferase data.

### 2.12. Statistical Analysis

All the data were included for statistical analyses using GraphPad Prism 8. The unpaired Student t-test (two-tailed) was used for the comparison between two unpaired groups. One-way analysis of variance (ANOVA) was applied for multi-group data comparison. Bar graphs were presented as means ± s.d. Differences were considered statistically significant at *p* < 0.05.

## 3. Results

### 3.1. GO and KEGG Analysis Indicated the Involvement of miRNAs in Lipid Storage and Fatty Acid Metabolism

**Figure 2:**
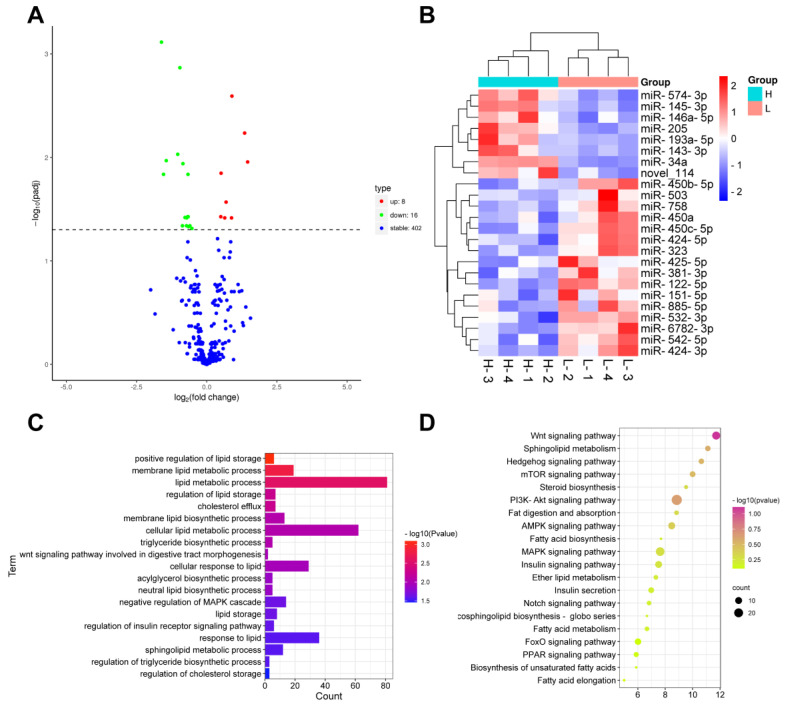
DE miRNAs significantly enriched in lipid storage and fatty acid metabolism signaling pathways. Volcano plot (**A**) demonstrated 8 upregulated miRNAs and 16 downregulated miRNAs (**B**) between high and low IMF-content muscles. GO (**C**) and KEGG (**D**) analysis indicated the enrichment of lipid storage and metabolism signaling pathways.

According to our previous studies [23], we classified the samples into two groups based on fat content: the high intramuscular fat group (H group, IMF content > 12%) and low intramuscular fat group (L group, IMF content < 5%), with 4 samples in each group. Then, mRNA and miRNA-sequencing were performed and analyzed to investigate the DE mRNAs and DE miRNAs profiles. In total, 24 DE miRNA were found, including 8 upregulated miRNAs and 16 downregulated miRNAs (Figure 2A,B). GO analysis showed that the DE miRNAs significantly enriched in positive regulation of lipid storage, lipid metabolic process, cholesterol efflux, and cellular response to lipid (Figure 2C). Moreover, KEGG analysis also demonstrated the enrichment of the Wnt signaling pathway, mTOR signaling pathway, AMPK signaling pathway, and PPAR signaling pathway (Figure 2D).

**Figure 3:**
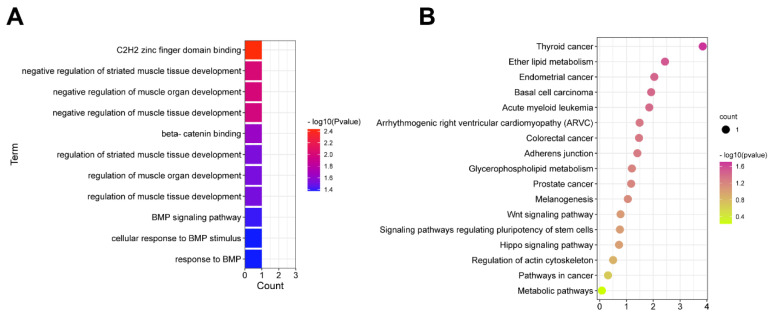
Functional enrichment analysis of co-expressed miRNA–mRNA network. GO (**A**) and KEGG (**B**) analysis indicated the involvement of *miR-34a*/*Lef1* in fat deposition.

Then, the co-expression pattern and Pearson correlation analysis were performed to determine the DE miRNA–mRNA potential regulatory networks. Interestingly, the results indicated that three DE miRNA have significant correlation with DE mRNA, including *miR-34a*-*Lef1*, miR-532-3p-PLD3, HMCES, miR-6782-3p-ARHGEF7, JARID2, and DDX54. According to GO and KEGG analysis, we found *Lef1* mainly enriched in the muscle and adipose tissue development (Figure 3A,B). Therefore, we suspect that *miR-34a*/*Lef1* may play an important role in regulating IMF accumulation.

### 3.2. miR-34a and Lef1 Show Opposite Expression Patterns during the Differentiation of 3T3-L1-Derived Adipocytes

**Figure 4:**
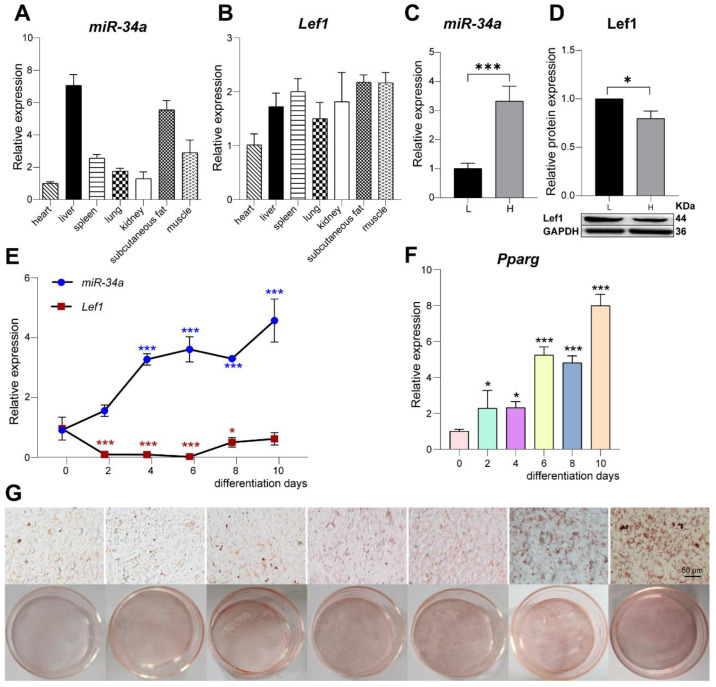
The expression pattern of *miR-34a* and *Lef1* in different tissues and the differential process of 3T3-L1-derived adipocytes. *miR-34a* are highly expressed in liver, backfat, and muscle (**A**), and *Lef1* was highly expressed in spleen, backfat and muscle (**B**). qPCR indicated *miR-34a* expression was higher in high IMF-content muscle (**C**), while *Lef1* protein was lower in high IMF-content muscle detected using Western blot (**D**). The expression of *miR-34a* was gradually increased with the differentiation of 3T3-L1-derived adipocytes, while *Lef1* was decreased (*n* = 4) (**E**). The expression of *Pparg*G (*n* = 4) and lipid droplet accumulation were gradually increased during the differentiation of 3T3-L1 cells (**F**,**G**). The expression of genes or proteins were normalized to the corresponding housekeeping gene or protein (β-actin and U6 for qPCR and GAPDH for Western blot). * *p* < 0.05, *** *p* < 0.001 versus Control.

To verify the expression of *miR-34a* and *Lef1* in the tissues, total RNA was extracted from the heart, liver, spleen, lung, kidney, subcutaneous fat, and muscle of each Laiwu pig from the H and L groups. The results show that these two genes were commonly expressed in these tissues. Additionally, *miR-34a* was highly expressed in liver, muscle and backfat, and *Lef1* was highly expressed in spleen, backfat, and muscle (Figure 4A,B). However, *miR-34a* expression was upregulated in the high IMF-content group (*p* < 0.01), while the Lef1 protein level in H group was significantly lower than that in the L group (*p* < 0.05) (Figure 4C,D). In order to explore the expression patterns of *miR-34a* and *Lef1* during preadipocyte differentiation, 3T3-L1 cells were induced to differentiate into adipocytes (d 0–d 10). With the differentiation degree of adipocytes, the expression of *Pparg* and lipid droplet accumulation were gradually increased (Figure 4F,G). Interestingly, the expression of *miR-34a* was gradually upregulated (*p* < 0.05), which is consistent with the expression pattern in muscle tissue (Figure 4E), while *Lef1* expression was significantly decreased at the early stage of differentiation (d 0–d 8) (*p* < 0.05) and increased subsequently (Figure 4E). Taken together, *miR-34a* and *Lef1* show opposite expression during the differentiation of 3T3-L1-derived adipocytes, indicating the potential regulatory interaction.

### 3.3. Lef1 Is a Target of miR-34a

**Figure 5:**
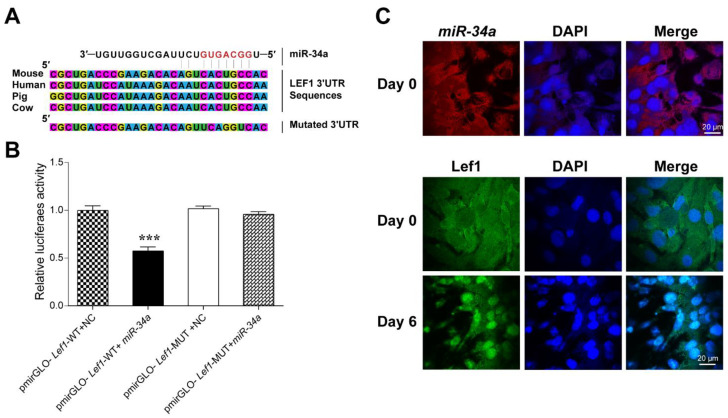
The targeting relationship and cellular location of *miR-34a* and *Lef1*. (**A**) The targeting relationship predicted with TargetScan and Starbase websites. (**B**) Luciferase reporter assay was performed to detect luciferase activity in HEK293 cells co-transfected with pmirGLO-*Lef1*-WT or pmirGLO-*Lef1*-MUT reporter and *miR-34a* mimics or NC. RNA FISH (**C**) indicated that miRA-34a was located in cytoplasm. *Lef1* was transferred into the nucleus with the differentiation of 3T3-L1-derived adipocytes (0 d–6 d). *** *p* < 0.001 versus NC (*n* = 3).

Based on the opposite expression pattern of *miR-34a* and *Lef1*, we hypothesized that *Lef1* may be a potential target of *miR-34a*. According to the prediction of the TargetScan and Starbase websites, *miR-34a* was well conserved in multiple species (mice, humans, pigs and cattle). Meanwhile, the seed sequence of *miR-34a* was complementary to the 7 nt base in the 3 ′UTR of *Lef1* (Figure 5A), which indicates that *miR-34a* may target *Lef1* to regulate IMF deposition. To confirm this hypothesis, *Lef1* wild-type (WT) or mutant (MUT) luciferase reporter vectors containing *miR-34a* binding sites were co-transfected with *miR-34a* or the NC into HEK293T cells. The luciferase reporter assay demonstrated that *miR-34a* overexpression effectively reduced luciferase activity of the pmirGLO-*Lef1*-WT reporter but did not decrease that of the pmirGLO-*Lef1*-MUT reporter (Figure 5B). These results suggest that mutation of the conserved 7 nt seed sequence abrogated the repression induced by *miR-34a* of the *Lef1* 3′UTR, demonstrating a targeted relationship between these two genes.

Then, RNA FISH assay and immunofluorescence staining were conducted to detect these respective gene expression locations. The results demonstrate that *miR-34a* and *Lef1* were mainly expressed in the cytoplasm. Interestingly, after 3T3-L1 cell differentiation, *Lef1* was found to be located in the nucleus (Figure 5C).

### 3.4. miR-34a Promoted Adipogenesis

**Figure 6:**
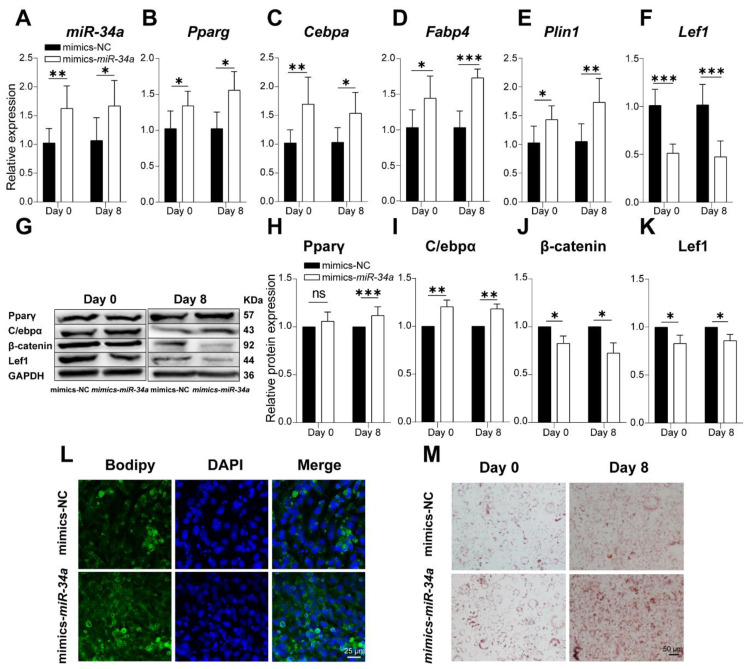
*miR-34a* overexpression promoted adipogenesis. (**A**) qPCR confirmed that 3T3-L1 cells stably overexpressed *miR-34a* after mimics treatments (*n* = 6). The expression of *Pparg* (**B**), *Cebpa* (**C**), *Fabp4* (**D**), *Plin1* (**E**), and *Lef1* (**F**) on day 0 and day 8 during 3T3-L1-derived preadipocyte differentiation after *miR-34a* mimics (*n* = 6). The protein expression level of Pparγ (**G**), C/ebpα (**H**), β-catenin (**I**), and Lef1 (**J**) in NC and *miR-34a* mimics groups along with differentiation via Western blot (*n* = 3) (**K**). Lipid droplet accumulation in 3T3-L1 cells on day 0 and 8 of differentiation detected using BODIPY staining (**L**) and Oil Red O staining (**M**) in NC and *miR-34a* mimics groups. * *p* < 0.05, ** *p* < 0.01, *** *p* < 0.001 versus NC.

To detect the effects of *miR-34a* on lipid deposition in adipocytes, *miR-34a* mimics and their control (mimics NC) were transfected into 3T3-L1 cells. Then, adipogenic markers (*Pparg*, *Cebpa*, *Fabp4,* and *Plin1*) and *Lef1* were detected during 3T3-L1 cell differentiation. Meanwhile, β-catenin, a specific and crucial molecule in the Wnt signaling pathway [31], was also detected in this study. As a result, *miR-34a* expression was successfully upregulated in 3T3-L1 cells following *miR-34a* mimic transfection (Figure 6A, *p* < 0.05). After overexpressing *miR-34a*, the expressions of *Pparg*, *Cebpa*, *Fabp4*, and *Plin1* were also upregulated compared with the NC group (*p* < 0.05). However, *miR-34a* mimics significantly reduced *Lef1* expression with preadipocytes differentiation (Figure 6B–F). In addition, the Western blot results show that *miR-34a* mimics significantly promoted the protein expression of PPARγ and C/EBPα and decreased Lef1 and β-catenin levels at d 0 and d 8 differentiation (Figure 6G–K). At the same time, the formation and accumulation of lipid droplet was promoted by overexpressing *miR-34a* detected via BODIPY (d 8) and oil red O staining (d 0 and d 8) (Figure 6L,M). These results indicated that overexpression *miR-34a* regulated the adipogenesis-associated molecules, promoting the accumulation of lipid droplets.

**Figure 7:**
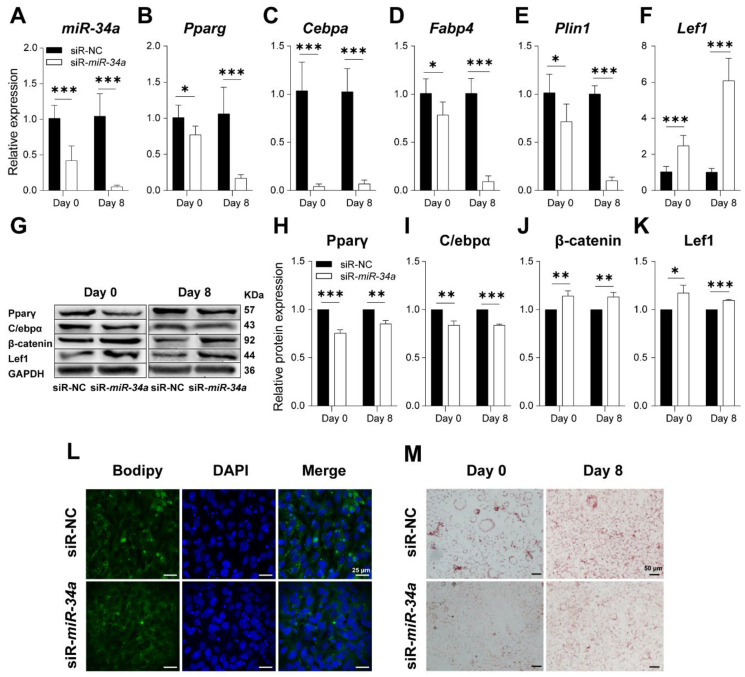
Silencing *miR-34a* suppresses adipogenesis. *miR-34a* inhibitor (siRNA) could efficiently decrease the expression of *miR-34a* in 3T3-L1 (*n* = 6) (**A**). The expression of *Pparg* (**B**), *Cebpa* (**C**), *Fabp4* (**D**), *Plin1* (**E**), and LEF1 (**F**) on day 0 and day 8 during 3T3-L1-derived preadipocyte differentiation after *miR-34a* silencing (*n* = 6). The protein expression level of Pparγ (**G**), C/ebpα (**H**), β-catenin (**I**), and Lef1 (**J**) in NC and *miR-34a* inhibitor groups along with differentiation via Western blot (*n* = 3) (**K**). Lipid droplet accumulation in 3T3-L1 cells on day 0 and 8 of differentiation detected using BODIPY staining (**L**) and Oil Red O staining (**M**) in NC and *miR-34a* inhibitor groups. * *p* < 0.05, ** *p* < 0.01, *** *p* < 0.001 versus NC.

To figure out the exact relationship between *miR-34a* and key adipogenic genes, *miR-34a* interference was performed. As can be seen from Figure 7A, *miR-34a* inhibitor successfully decreased *miR-34a* expression with the differentiation of 3T3-L1 cells (*p* < 0.001). The results of qPCR and Western blot demonstrate that *miR-34a* inhibitor significantly reduced the RNA and protein expression of Pparγ, C/ebpα, Fabp4, and Plin1 (Figure 7B–F). Meanwhile, the protein levels of PPARγ and C/EBPα were also decreased; however, the LEF1 and β-catenin protein expression was dramatically increased (*p* < 0.05) during the differentiation of 3T3-L1 cells into adipocytes (Figure 7G–K). Moreover, BODIPY and Oil Red O staining were also conducted to visually reveal the effect of *miR-34a* inhibitor on lipid droplet deposition in 3T3-L1 cells. According to Figure 7L,M, the deposition of lipid droplets in 3T3-L1 cells decreased after *miR-34a* expression was inhibited. Taken together, these results confirmed that silencing *miR-34a* could downregulate adipogenic genes, thereby suppressing the accumulation of lipid droplets during 3T3-L1-derived adipocyte differentiation.

### 3.5. miR-34a Promoted Fat Deposition by Suppressing Lef1 during 3T3-L1 Differentiation

**Figure 8:**
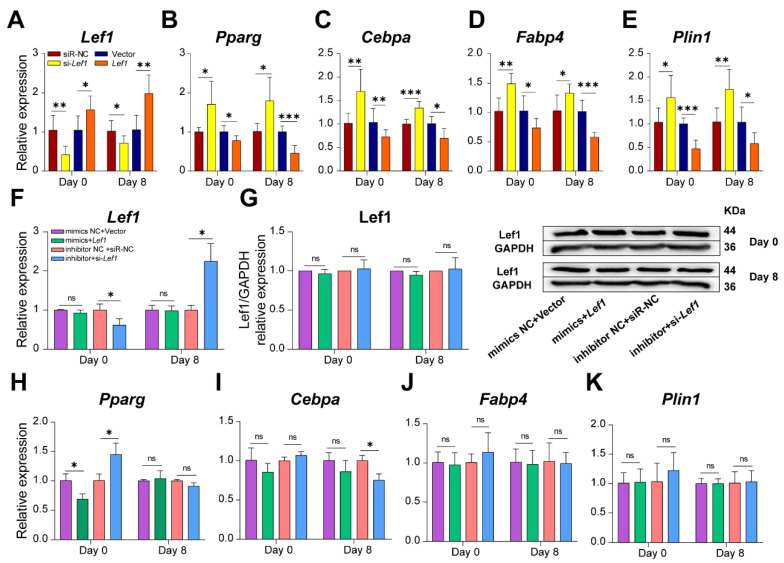
*miR-34a* abolished the suppressive function of overexpressed *Lef1* on fat deposition during 3T3-L1 differentiation. The expression of *Lef1* (**A**), *Pparg* (**B**), *Cebpa* (**C**), *Fabp4* (**D**), and perilipin1 (**E**) after cells transfected with pcDNA3.1-*Lef1*, si*Lef1* and their corresponding control during cell differentiation (*n* = 6). The expression of *Lef1* (**F**), *Pparg* (**H**), *Cebpa* (**I**), *Fabp4* (**J**), and perilipin1 (**K**) after cells transfected with *miR-34a* mimics NC + pcDNA3.1 or mimics + pcDNA3.1-*Lef1* or with *miR-34a* inhibitor NC + siNC or inhibitor + si*Lef1* during cell differentiation (*n* = 3). The protein expression of Lef1 during cell differentiation in the rescue experiment (*n* = 3) (**G**). * *p* < 0.05, ** *p* < 0.01, *** *p* < 0.001 versus NC.

In order to investigate the function of Lef1 in the process of fat deposition in 3T3-L1 cells, adipogenic-associated gene expression levels were detected after overexpressing or silencing *Lef1*. PcDNA3.1-vector-containing *Lef1* and *Lef1* siRNA could significantly upregulate or silence the expression of *Lef1* (Figure 8A). *Lef1* overexpression significantly decreased the levels of *Pparg*, *Cebpa*, *Fabp4,* and *Plin1*; however, when the *Lef1* expression was disturbed, the adipogenic markers were dramatically upregulated (*p* < 0.05) (Figure 8B–E). These results suggest that *Lef1* negatively regulated lipid deposition during the differentiation of 3T3-L1 cells.

To further explore whether the effects of *miR-34a* on lipid accumulation were mediated by *Lef1*, we conducted a rescue experiment by simultaneous overexpressing or inhibiting *miR-34a* and *Lef1* in 3T3-L1 cells by transfecting *miR-34a* mimics/inhibitor or pcDNA3.1-NC/*Lef1*. As is shown in Figure 8F,G, the overexpression caused by transfecting pcDNA3.1-*Lef1* was rescued by *miR-34a* mimics during 3T3-L1 differentiation on day 0 or day 8. Meanwhile, the protein expression of *Lef1* also showed no difference after co-transfection of *miR-34a* and *Lef1* to preadipocytes. However, when cells were treated with *miR-34a* inhibitor plus si*Lef1*, *Lef1* expression was significantly decreased (Figure 8F), while the *Pparg* level was significantly increased before 3T3-L1 cell differentiation (*p* < 0.05, Figure 8H). After 3T3-L1 cell differentiation at d 8, *Lef1* expression was significantly increased, but the *Cebpa* level was significantly decreased in the group of *miR-34a* inhibitor plus si*Lef1* (*p* < 0.05, Figure 8I). In addition, there was no significant difference in the expression of adipogenic markers in the four treatments during 3T3-L1 cell differentiation. These results suggest that the overexpression of *miR-34a* reversed the inhibitory function of overexpressed *Lef1* on lipid deposition in 3T3-L1 cells.

### 3.6. miR-34a/Lef1 Promoted Lipid Droplet Accumulation in C2C12 Cells

**Figure 9:**
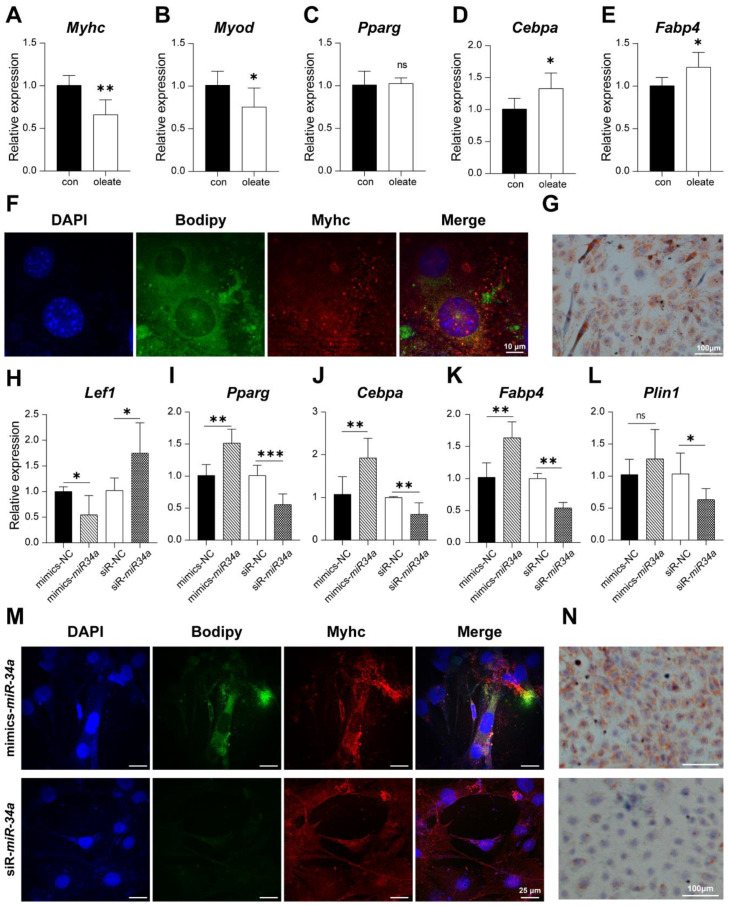
*miR-34a* positively regulates the expression of adipogenic genes in C2C12 cells. The expression of My*hc* (**A**), *Myod* (**B**), *Pparg* (**C**), *Cebpa* (**D**), and *Fabp4* (**E**), as well as immunofluorescence (**F**) and Oil Red O staining (**G**) assays when C2C12 induced by oleate for 24 h. The expression of *Lef1* (**H**), *Pparg* (**I**), *Cebpa* (**J**), *Fabp4* (**K**), and *Plin1* (**L**), as well as immunofluorescence (**M**) and Oil Red O staining (**N**, n = 3), when C2C12 cells were transfected with the miR-34a mimics, inhibitor, or their corresponding controls. The scale bar of immunofluorescence was 25 μm and the magnification was 4×. The qPCR data showed the means of six independent experiments. * *p* < 0.05, ** *p* < 0.01, *** *p* < 0.001 versus NC.

Different types of cells might have interactions in location microenvironments, thus we suspect that the fat deposition process of adipocytes might affect muscle cell metabolism. Previous studies have found that oleate can promote lipid droplet accumulation in C2C12 cells [28]. Based on this study, the model of lipid droplet deposition in myoblasts was constructed. As shown in Figure 9A–E, 500 μM oleate significantly decreased myogenesis marker (MyHC and MyOD) expression but promoted the expression of *Fabp4* and *Cebpa* (*p* < 0.05). In addition, BODIPY and Oil Red O staining showed that 500 μM oleate could promote the accumulation of lipid droplets in C2C12 cells (Figure 9F,G). Thus, the model of fat deposition in myoblasts induced by oleate was established successfully.

Then, qPCR was performed to investigate whether *miR-34a* could affect the lipid droplet deposition in C2C12 cells induced by oleate. The results show that overexpression of *miR-34a* significantly inhibited the expression of *Lef1* (*p* < 0.05) (Figure 9H). Meanwhile, the overexpression of *miR-34a* significantly promoted the expression of *Pparg*, *Cebpa,* and *Fabp4* in C2C12 cells induced by oleate (*p* < 0.05) but had no significant effect on *Plin1* (Figure 9I–L). When *miR-34a* expression was disrupted, *Lef1* expression was significantly increased (*p* < 0.05); however, the expression of *Pparg*, *Cebpa*, *Fabp4,* and *Plin1* was significantly decreased (*p* < 0.05). To better reveal the effect of *miR-34a* on lipid droplet deposition in C2C12 cells, BODIPY and Oil Red O staining were performed after the cells were induced by oleate. These results showed that *miR-34a* mimics promoted the accumulation of lipid droplets in myoblasts (Figure 9M,N). However, *miR-34a* inhibitor showed the opposite function.

### 3.7. miR-34a Increased Fat Deposition in 3T3-L1 and C2C12 Cells Co-Culture System

**Figure 10:**
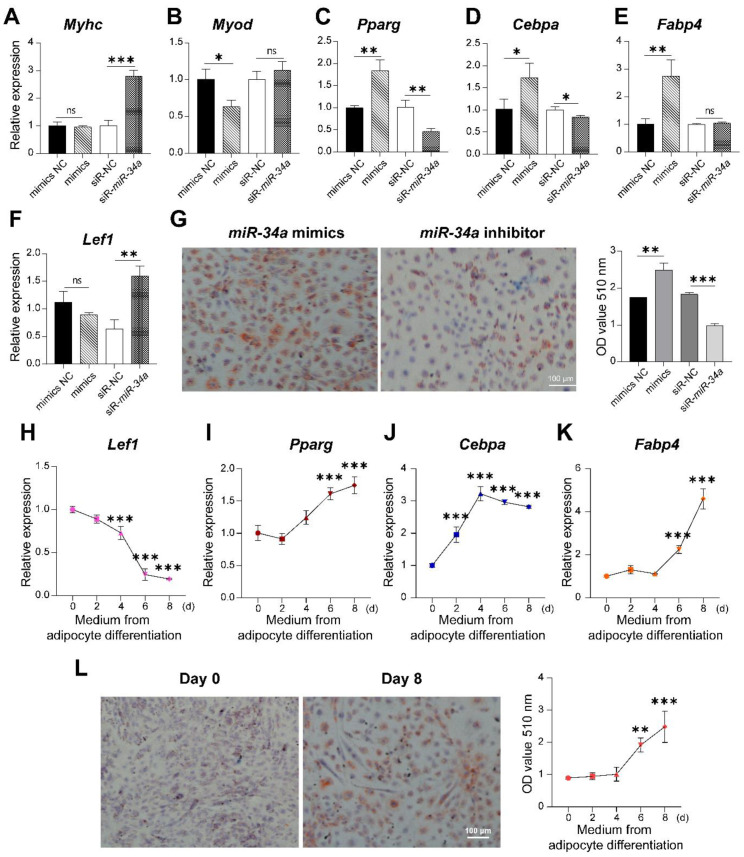
*miR-34a*/*Lef1* regulated intramyocellular lipid deposition in co-culture system and C2C12 cells cultured with conditioned medium. The expression of MyHC (**A**), MyOD (**B**), *Pparg* (**C**), *Cebpa* (**D**), *Fabp4* (**E**), and *Lef1* (**F**), as well as Oil Red O staining [28] (**G**) in the co-culture of C2C12 and 3T3-L1 cells transfected with *miR-34a* mimics or inhibitor. The levels of *Lef1* (**H**), *Pparg* (**I**), *Cebpa* (**J**), *Fabp4* (**K**), as well as Oil Red O staining (**L**) in the C2C12 cells incubated in conditioned medium with different times of adipocyte differentiation. The magnification was 4×. The qPCR data showed the means of six independent experiments and the data of OD value (510 nm) showed the means of three independent experiments. * *p* < 0.05, ** *p* < 0.01, *** *p* < 0.001 versus NC.

Generally, fat deposition in adipocytes distributed in muscle tissue is the primary reason for intramuscular fat deposition. However, we found *miR-34a* can promote the lipid droplets deposition in C2C12 cells. Thus, we suspect that the IMF accumulation in muscle might be the co-contribution of adipocytes and muscle cells. Therefore, the co-culture model of C2C12 and 3T3-L1 cells was constructed to detect whether there is an interaction between these two types of cells. *miR-34a* mimics, inhibitor, and their corresponding controls (mimics NC or inhibitor NC) were transfected into the co-cultured cells. Results, as shown in Figure 10A–F, indicate that *miR-34a* mimics significantly promoted the expression of *Pparg*, *Cebpa,* and *Fabp4* and decreased MyOD expression (*p* < 0.05). While *miR-34a* inhibitor upregulated the expression of MyHC and *Lef1* and repressed adipogenic marker levels (*p* < 0.05), Oil Red O staining showed that *miR-34a* overexpression increased the accumulation of lipid droplets, and the OD value of Oil Red O extraction with isopropanol in the *miR-34a* mimics treatment was significantly increased compared with the NC group (*p* < 0.05).

### 3.8. miR-34a Increased IMF Deposition in C2C12 Cells Incubated with the Adipogenic Conditioned Medium

According to the function of *miR-34a* in the co-culture of 3T3-L1 and C2C12 cells, it is speculated that adipocytes have a relationship with the lipid accumulation in myoblasts through intercellular communication. Therefore, we transfected *miR-34a* mimics into 3T3-L1 cells. Then, the supernatant during preadipocyte differentiation (d 0–d 8) was collected and used as conditioned medium for the culture of C2C12 cells after C2C12 cell differentiation. The results show that with the preadipocytes’ differentiation, the conditioned medium significantly inhibited *Lef1* expression and promoted the levels of adipogenic markers (Figure 10H–K, *p* < 0.05). Oil Red O staining on d 8 in conditioned medium was significantly increased compared with the d 0 group (*p* < 0.05) (Figure 10L).

## 4. Discussion

IMF (marbling) is an essential index for pork quality in terms of tenderness and juiciness. The content of it is mainly associated with the number and size of intramuscular adipocytes [32]. The IMF content of Laiwu pigs was higher than that of other pigs. To date, most studies selected the DEGs related to IMF deposition through comparisons between Laiwu pigs and other breeds of pig [33], but few have focused on variation among individuals belonging to the same breed. Our previous study demonstrated that the IMF content of individual Laiwu pigs varies greatly (about 2.17–13.93%) [23], which has been replicated in other studies over the past year [22]. The knowledge regarding this phenomenon is still in its infancy. This study is the first to propose why there are differences in IMF deposition among different individual pigs of the same breed.

The *miR-34a* family consists of *miR-34a*, miR-34b, and miR-34c. *miR-34a* is commonly expressed in multiple tissues, while miR-34b and miR-34c are mainly expressed in the lungs [34]. This study found that *miR-34a* was highly expressed in liver, subcutaneous fat, and muscle tissues of Laiwu pigs. Evidence has shown that *miR-34a* is significantly elevated in the liver of obese mice [35] and upregulated during adipogenesis [36]. These results suggest that *miR-34a* may play an important role in IMF deposition. There are multiple target genes for *miR-34a*, and SIRT1 is the most studied target gene at present [20,21,37]. In addition, *miR-34a* could target PDGFRα to regulate the differentiation of intramuscular preadipocytes through the ERK signaling pathway [38]. However, the mechanisms of *miR-34a* regulating porcine IMF deposition by targeting *Lef1* have not been reported yet.

Mature miRNAs mainly exist in the cytoplasm, and current studies show that they are also distributed in the nucleus, mitochondria, and exosomes [39,40,41]. Therefore, the expression and localization of miRNAs in cells is the basis for studying their functions. Our results show that *miR-34a* and *Lef1* were mainly expressed in the cytoplasm. This is consistent with the general environment in which the relationship between miRNA and mRNA operates. Studies have shown that the transcription factor Lef1 can transfer into the nucleus and bind with β-catenin to regulate downstream gene expression [42]. Our results in this study also confirm that *Lef1* was transferred into the nucleus after preadipocyte differentiation. According to the analysis of TargetScan and Starbase, the targeted binding site of *miR-34a* and *Lef1* was predicted, which was confirmed by the dual luciferase reporter assay. In addition, during preadipocyte differentiation, *Lef1* expression was negatively correlated with *miR-34a* at the early stage of differentiation, but this negative correlation is not strict at the late stage of differentiation. There was evidence to explain this phenomenon. The interaction between miRNAs and their target genes is not a constant negative correlation but a dynamic biological change process under different environments [12]. miRNAs could activate translation of targeted mRNAs, which may be triggered by changes in growth microenvironment [43].

IMF is an essential index for the tenderness and juiciness of pork. Studies have shown that the growth and development of skeletal muscle and adipocytes take an important role in meat quality [44]. Meanwhile, the co-culture results of C2C12 and 3T3-L1 cells show that the interaction between these two cells regulated the expression of calprotease, caspase, and heat shock protein in meat [45]. Considering the complexity and particularity of the IMF microenvironment in vivo, the function of *miR-34a* and LEF1 in myoblasts and adipocytes is of significance to explore the mechanisms of IMF deposition. Therefore, this study constructed a co-culture model of C2C12 and 3T3-L1 cells to explore the function of *miR-34a* and *Lef1* in intercellular communication between adipocytes and myoblasts during IMF accumulation. Meanwhile, C2C12 cells were cultured in conditioned medium collected during 3T3-L1 cell differentiation to study the *miR-34a* and *Lef1* effects of adipocyte secretion on lipid deposition in myoblasts.

**Figure 11:**
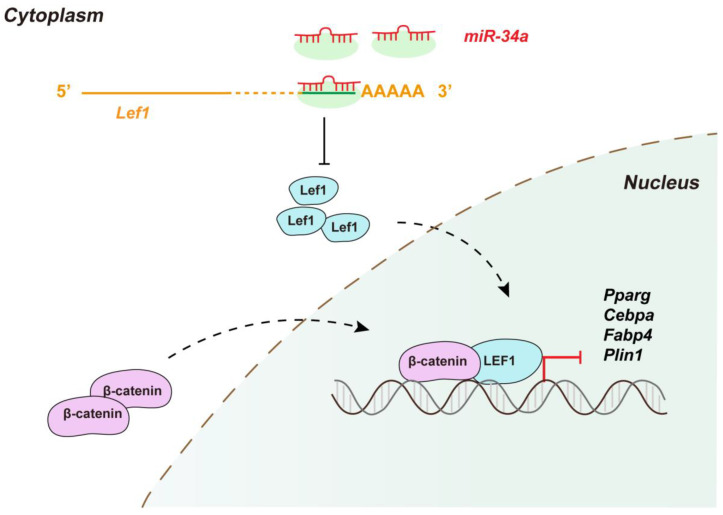
The process of *miR-34a*/*Lef1* regulating adipogenesis. *Lef1* transferred to nucleus and combined with β-catenin to suppress the transcription of adipogenic markers, such as *Pparg*, *Cebpa*, *Fabp4,* and *Plin1*, while the suppressive function of *Lef1* can be inhibited by *miR-34a*.

First, *miR-34a* mediated *Lef1* to regulate lipid droplet deposition in 3T3-L1 cells. Lef1 is the factor at the end of the Wnt signaling pathway. The dimer of Lef1 and β-catenin regulate the expression of downstream transcription factors after the Wnt signaling pathway is activated, playing an important part in myogenesis and adipogenesis [46]. In this study, *miR-34a* inhibited the expression of *Lef1* and β-catenin and upregulated the levels of *Pparg*, *Cebpa*, *Fabp4,* and *Plin1* (adipogenic markers) during preadipocyte differentiation. In addition, the rescue test showed that the overexpression of *miR-34a* could reverse the inhibition of *Lef1* on adipogenic markers in 3T3-L1 cells. These results suggest that *miR-34a* regulated IMF deposition by targeting *Lef1* (Figure 11). This was consistent with previous studies on *Lef1*/β-catenin (Wnt signaling pathway) regulating fat deposition in obesity [47].

*miR-34a* mediated *Lef1* to regulate lipid droplet deposition in C2C12 cells. The lipid droplets’ accumulation in myoblasts is an important aspect to enhance the IMF content. Evidence shows that BMP11 could negatively regulate lipid metabolism in C2C12 cells, inhibit myogenesis, and promote fatty acid accumulation and lipid droplet formation [48]. VEGF could upregulate the expression of fatty acid transporters FATP1 and FATP4 and promote fatty acid oxidation and TG decomposition in C2C12 cells, thereby inhibiting lipid accumulation in C2C12 cells [49]. Studies showed that oleate could induce lipid deposition in skeletal muscle cells [28]. Therefore, oleate was used to induce lipid droplet deposition in myoblasts. The results demonstrate that *miR-34a* promoted the levels of adipogenic markers in C2C12 cells by inhibiting *Lef1* expression.

*miR-34a* regulated lipid droplet deposition in the co-culture of C2C12 and 3T3-L1 cells by mediating *Lef1*. Evidence shows that the co-culture of C2C12 and 3T3-L1 cells could be used to simulate the construction of muscle and fat formation [50]. The co-culture of 3T3-L1 and RAW264.7 cells was used to investigate the inflammatory regulation of obesity and macrophage infiltration [51]. This study showed that *miR-34a* overexpression decreased MyOD expression, while the inhibition of *miR-34a* significantly increased the MyHC level. These results suggest that *miR-34a* could reduce myogenic marker expression to inhibit C2C12 cell differentiation. Similar to this result, 3T3-L1 cells inhibit the differentiation of C2C12 cells by promoting the expression of myostatin in the co-culture system [52]. Meanwhile, this study found that *miR-34a* overexpression significantly promoted adipogenic markers expression and increased lipid droplet accumulation in the co-culture of C2C12 and 3T3-L1 cells. However, after *miR-34a* was inhibited, *Lef1* expression was significantly increased, while the levels of adipogenic markers and lipid droplet deposition were decreased. These results suggest that *miR-34a* inhibited myoblast differentiation and promoted lipid deposition in the co-culture of C2C12 and 3T3-L1 cells by targeting *Lef1*.

*miR-34a* mediated *Lef1* to indirectly regulate lipid deposition in myoblasts through influencing the adipocytes secretion. According to the *miR-34a* regulation on lipid deposition in the co-culture cells, it was speculated that *miR-34a* could regulate lipid droplet accumulation in myoblasts through secretions during preadipocyte differentiation. Therefore, *miR-34a* mimics were transfected into 3T3-L1 cells. During preadipocyte differentiation, the supernatant was collected and prepared as a conditioned medium for C2C12 culture after filtration. In this study, *Lef1* expression was significantly inhibited and the expression of adipogenic markers and lipid droplet deposition in C2C12 cells was promoted in the conditioned medium. These results suggest that *miR-34a* mediated *Lef1* to regulate lipid deposition in myoblasts by influencing secretions during preadipocyte differentiation.

In this study, based on the results of the co-culture and conditioned medium treatments, the hypothesis that the secretion of adipocytes may play an important role in IMF accumulation can be established. Previous studies used the supernatant from adipocytes of different degrees of hyperplastic obesity for culture, which was absorbed and filtered to prepare a conditioned medium for preadipocytes culture, and this conditioned medium promoted preadipocyte differentiation [53]. Exosomes are actively exfoliated endontic vesicles, approximately 40 to 100 nm in diameter, which contain and transport functional mRNAs, miRNAs, and proteins between different cells [54,55]. Exosomes derived from adipocytes can induce obesity by enhancing lipid deposition in macrophages and increasing pro-inflammatory cytokines, thereby leading to insulin resistance [56]. Meanwhile, miRNAs in exosomes derived from adipocytes mediate TGF-β and Wnt signaling pathways to regulate adipogenesis [57]. miR-199a-5p in exosomes derived from muscle fibroblasts promote the fibrosis of skeletal muscle [58]. Therefore, the reasons for *miR-34a* regulation of lipid deposition in the co-culture and conditioned medium treatment were as follows: *miR-34a* may be secreted by adipocytes to regulate lipid droplet deposition in myoblasts, and *miR-34a* targets *Lef1* to regulate the secretion factors of adipocytes during preadipocyte differentiation and then induces lipid droplet deposition in C2C12 cells. However, how *miR-34a* and *Lef1* regulate lipid accumulation by changing secretions during preadipocyte differentiation remains to be further explored.

## 5. Conclusions

In conclusion, due to the particularity and complexity of the microenvironment of IMF in vivo, fat deposition in adipocytes and myoblasts, signal exchange between myoblasts and preadipocytes, and the regulation of the secretion of adipocytes in IMF deposition were discussed in this study. We found that *miR-34a* promoted fat deposition in adipocytes and myoblasts by targeting *Lef1* through the Wnt (Lef1/β-catenin) signaling pathway. Meanwhile, *miR-34a* targeting *Lef1* could promote IMF deposition by regulating signal exchange between myoblasts and adipocytes. In addition, *miR-34a* mediated *Lef1* to regulate lipid droplet deposition in myoblasts through secretions collected from preadipocyte differentiation. Therefore, this study confirmed that *miR-34a* can regulate IMF deposition by targeting *Lef1*. However, the mechanism of how *miR-34a* indirectly regulates lipid droplet accumulation in myoblasts through adipocyte secretions remains unclear. In addition, more research should be conducted with a pig model to verify the conclusion of our study, which is also a limitation of our study.

## Figures and Tables

**Table 1:** Primers for qPCR analysis and the sequence of *miR-34a* mimics/inhibitor and si-*Lef1* and their negative controls.

Gene	Accession Number	Species	Sequence (5′-3′)
*Myhc*	AH002050	mouse	F: GGAGCAGACGGAGAGGAGCAG
			R: TTGGTGTTGATGAGGCTGGTGTTC
*Myod*	M84918	mouse	F: TCCAACTGCTCTGATGGCATGATG
			R: ACTGTAGTAGGCGGTGTCGTAGC
*Plin1*	NM_175640	mouse	F: GCACAACCTGGCAGCCTCTC
			R: CTTCCTCCTCCTCGTCGTCTGTC
*Fabp4*	CT010390	mouse	F: AAATCACCGCAGACGACAGGAAG
			R: CATTCCACCACCAGCTTGTCACC
*Cebpa*	NM_001287523	mouse	F: GAGGGGAGGGACTTAGGTGTTGG
			R: CCTGGCCTGTTGTAAGCTGAGTG
*Lef1*	NM_010703	mouse	F: CAACTAGAAAGCCAGCGACCAGAG
			R: GGAAAATGAAGACAGGAGCGAGAGG
*LEF1*	NM_001129967	sus scrofa	F: GGGTCGGGCTGTGTGTGTTTC
			R: CTCAGGGAGTCGGCAGTGGAG
*Pparg*	CT010340	mouse	F: GTTCGCCAAGGTGCTCCAGAAG
			R: GTGAAGGCTCATGTCTGTCTCTGTC
*PPAR* *G*	NM_214379	sus scrofa	F: TCTGTGGACCTGTCGGTGATGG
			R: TCAGCTCTCGGGAATGGGATGTC
*β-actin*	AY618569	mouse	F: CAGATGTGGATCAGCAAGCAGGAG
			R: CAGTAACAGTCCGCCTAGAAGCAC
*β-ACTIN*	AJ312193	sus scrofa	F: AATCCTGCGGCATCCACGAAAC
			R: CAGCACCGTGTTGGCGTAGAG
*miR-34a*	NR_029751	mouse/sus scrofa	F: CTGGCAGTGTCTTAGCTGGTTGTT
*U6*	NM_015816	mouse/sus scrofa	F: ACGATACCCGCAAGGATGACA
*miR-34a mimics*		mouse	F: UGGCAGUGUCUUAGCUGGUUGU
			R: AACCAGCUAAGACACUGCCAUU
*mimics* *NC*		mouse	F: UUCUCCGAACGUGUCACGUTT
			R: ACGUGACACGUUCGGAGAATT
*miR-34a* *inhibitor*		mouse	F: ACAACCAGCUAAGACACUGCCA
*inhibitor NC*		mouse	F: CAGUACUUUUGUGUAGUACAA
*siLef1*		mouse	F: CGUCAGAUGUCAACUCCAATT
			R: UUGGAGUUGACAUCUGACGTT
*siNC*		mouse	F: UUCUCCGAACGUGUCACGUTT
			R: ACGUGACACGUUCGGAGAATT

## Data Availability

Raw RNA sequencing data have been submitted to the NCBI Sequence Read Archive under accession number PRJNA769962.
